# Biocarbons Obtained from Fennel and Caraway Fruits as Adsorbents of Methyl Red Sodium Salt from Water System

**DOI:** 10.3390/ma15228177

**Published:** 2022-11-17

**Authors:** Aleksandra Bazan-Wozniak, Dorota Paluch, Robert Wolski, Judyta Cielecka-Piontek, Agnieszka Nosal-Wiercińska, Robert Pietrzak

**Affiliations:** 1 Faculty of Chemistry, Adam Mickiewicz University in Poznań, Uniwersytetu Poznańskiego 8, 61-614 Poznań, Poland; 2 Department of Pharmacognosy, Poznan University of Medical Sciences, Rokietnicka 3, 60-806 Poznań, Poland; 3 Faculty of Chemistry, Maria Curie-Sklodowska University in Lublin, Maria Curie-Sklodowska 3, 20-031 Lublin, Poland

**Keywords:** biocarbon, fennel and caraway fruits, direct physical activation, methyl red sodium salt, kinetic and equilibrium study

## Abstract

The aim of this study was to prepare biocarbons by biomass activation with carbon(IV) oxide. Fennel and caraway fruits were used as the precursors of bioadsorbents. The impact of the precursor type and temperature of activation on the physicochemical properties of the obtained biocarbons and their interaction with methyl red sodium salt upon adsorption process have been checked. The obtained bioadsorbents were characterized by determination of-low temperature nitrogen adsorption/desorption, elemental analysis, ash content, Boehm titration, and pH of water extracts. The biocarbons have surface area varying from 233–371 m^2^/g and basic in nature with acidic/basic oxygen-containing functional groups (3.23–5.08 mmol/g). The adsorption capacity varied from 63 to 141 mg/g. The influence of different parameters, such as the effectiveness of methyl red sodium salt adsorption, was evaluated. The adsorption kinetics was well fitted using a pseudo-second-order model. The Freundlich model best represented the equilibrium data. The amount of adsorbed dye was also found to increase with the increasing temperature of the process.

## 1. Introduction

Over the past decades we have seen the deteriorating quality of drinking water, which is caused by rapid industrialization, ever-increasing population, urbanization and reckless use of natural resources, among other factors [1,2]. The amount of waste and number of toxic gases released into the environment is increasing year by year due to the continuous development of industry and progress of civilization. On the other hand, the standards for permissible emissions of pollutants are constantly tightened [3,4,5,6].

Organic substances, pharmaceuticals, personal care products, biocides, heavy metals, dyes, plastics, nanoparticles and pathogens are just some of the contaminants that pose a major problem [7,8,9,10]. One of the measures taken to protect the environment is to develop effective adsorbents that would exhibit high adsorption of liquid and gas pollutants.

It has been estimated that on average 10–15% of dyes enter wastewater during the textile dyeing process [11,12]. Most of them are considered non-biodegradable due to their molecular structure [13]. The presence of dyes has led to contamination of water bodies and reduced penetration of sunlight through the water sheet, which consequently contributes to serious changes in aquatic ecosystems. They are also hazardous to living organisms, causing mutagenic and carcinogenic changes [14].

Methyl red (MR) is commonly used in the textile sector and in microbiology as a pH indicator pH [15]. The wastewater containing methyl red is strongly colored and toxic. The toxic effect of dyes, including methyl red, on living organisms is due to their chemical structure and stability in the natural environment. Methyl red may provoke adverse reactions in the organisms through damage to blood vessels or inducing mutagenicity [15]. Therefore, it has been used in adsorption tests on porous materials [16,17,18,19]. Santhiet al. [16] have studied the applicability of activated carbon, obtained by activation of *Annonas qumosa* seeds by H_2_SO_4_, in the removal of MR from simulated wastewater. This carbon was shown to adsorb a maximum of 40.5 mg of methyl red. Ioannou et al. [17] have studied adsorption of MR on: zeolite, hematite, modified zeolite and commercial activated charcoal. According to the results presented in [17], the best material to adsorb methyl red was the commercial activated carbon. Only this adsorbent was able to ensure 100% effectiveness in removal of all the adsorbates studied. For the other three adsorbents, the effectiveness of methyl red removal varied from 10 to 65%. Ding et al. [18] have proved that biochar, which was the byproduct of gas production through a fast pyrolysis of sawdust, may be an effective adsorbent of methyl red, showing a high sorption capacity of 156.25 mg/g. The authors of [19] have reported that the effectiveness of methyl red adsorption on eggshells reached 82%, while the maximum sorption capacity of this adsorbent was 1.66 mg/g.

Several methods have been considered for removal of dyes from different solutions, such as precipitation, ion exchange, adsorption, and membrane separation [20,21,22]. However, the high cost of reagents needed, the formation of precipitate, and complicated handling can be considered as the main disadvantages of the aforementioned methods [21]. Adsorption is believed to have significant potential for the removal of synthetic dyes from water solution [23].

Adsorption processes with the employment of activated carbon are particularly effective. Due to its physicochemical properties, activated carbon is one of the most widely and commonly used adsorbents in pollutant removal processes [24,25,26]. The raw materials used in the preparation of activated carbons should be readily available, inexpensive, safe, and contain a high mass percentage of elemental carbon. The material should also have a low mineral content and show low biodegradability during storage. Popular activated carbon precursors include peat [27], wood [28] and fruit seeds [29]. Activated carbon can be successfully obtained from any natural waste material, such as fennel seeds or caraway seeds.

Physical activation is a cheap and fast process to obtain adsorbents, so the purpose of this study was to obtain biocarbons by direct physical activation of fennel and caraway fruits with carbon (IV) oxide and present their comprehensive physicochemical characterization. For the production of carbon adsorbents, we used microwave radiation, which permits heating of the whole volume of the sample. Moreover, the use of microwave radiation led to a decrease in the cost of biocarbon preparation relative to the method based on conventional heating (tubular furnace). The cost of the process was further reduced by application of direct physical activation, which is much cheaper than the usually used methods of activated carbons production [30]. The sorption capacity of the obtained biocarbons towards methyl red sodium salt was characterized. Freundlich and Langmuir adsorption isotherms were used to model the equilibrium adsorption data obtained for methyl red sodium salt adsorption on biocarbons made from two different raw materials. The influence of on their sorption capacities, as well as iodine adsorption numbers, were checked.

## 2. Material and Method

### 2.1. Precursors

The precursors of biocarbons studied were the fruit of fennel (*Foeniculum vulgare*) and caraway (*Carum carvi* L.). The raw materials (25 g each) were at first sieved with a mesh size of 0.7–1.0 mm, then washed with 20 L of distilled water and dried at 105 °C. The drying was continued for 48 h.

### 2.2. Direct Physical Activation

To minimize the cost of biocarbon preparation, the raw products were subjected to direct physical activation. In order to ensure homogeneous heating of the whole sample volume, the microwave oven was used (Phoenix, CEM Corporation, Matthews, IL, USA). The fruit of fennel (F) and caraway (C) were subjected to activation with carbon(IV) oxide (flow rate 200 mL/min, heating rate 10 °C/min) in two temperature variants: at 700 (A7) or 800 °C (A8) in a microwave oven for 30 min. After this process, the biocarbons were cooled down to room temperature in nitrogen flow in the microwave oven.

### 2.3. Biocarbons Characterization

Thermo Scientific FLASH 2000 Elemental Analyzer (OEA, Thermo Fisher Scientific, Waltham, MA, USA) was applied to establish the elemental composition of biocarbons. The standard test method for ash was performed according to the ASTM D2866-94 Standard (2004).

The prepared biocarbons were characterized for their porous properties using nitrogen adsorption/desorption isotherms measured at 77 K by using an analyzer AutosorbiQ instrument, provided by Quantachrome Instruments (Boynton Beach, FL, USA). Before adsorption measurements, the biocarbons were degassed under vacuum for 12 h at 300 °C. The specific surface area was calculated from the nitrogen adsorption isotherm data according to the Brunauer–Emmett–Teller method. The total pore volume (V_T_) was estimated from the volume of nitrogen adsorbed at the relative pressure of p/p_0_ = 0.99, which is the equilibrium pressure divided by the saturation pressure and converted to the volume of nitrogen in the liquid state at a given temperature. The average pore size (D) was calculated from the equation: D = 4V_t_/S_BET_. S_BET_ is the surface area, and the pores are assumed to have cylindrical shape.

The SEM images of biocarbons were obtained using the scanning electron microscope Quanta 3D FEG (FEI, Field Electron and Ion Co, Hillsboro, Oregon, USA.).

The pH values of each biocarbon and the content of oxygen functional groups were measured by the procedure described in papers [31,32]. Additionally, iodine adsorption analysis was performed for the obtained biocarbons [31].

### 2.4. Adsorption of Methyl Red Sodium Salt

The stock water solution of methyl red sodium salt (MR, 1000 mg/L) was made, from which solutions of desired concentrations from the range 10–80 mg/L were prepared as needed. To perform the adsorption study, 25 mg of each biocarbon were mixed with 0.050 L of a given dye solution of an appropriate concentration. The samples were shaken for 12 h on a shaker (Heidolph, Schwabach, Germany) at 200 rpm/min. Next, the solid adsorbents were separated from the solution using a laboratory centrifuge (OHAUS, Parsippany, NJ, USA). The concentration of methyl red sodium salt in the solution was determined spectrophotometrically at λ_max_ 443 nm, using a Carry 100 Bio spectrophotometer (Agilent, Santa Clara, CA, USA). The amount (q_e_) of MR adsorbed on 1 g of a given biocarbon was calculated from the Formula (1):

(1) $$q_{e}=\frac{C_{0}-C_{e}}{m}\times V$$

where: C_0_is the initial concentration of MR solution [mg/L]; C_e_is the concentration of MR remaining in solution at equilibrium [mg/L]; mis the mass of a biocarbon; andV is the volume of MR solution [L].

The effect of the pH (pH values 2–12) of methyl red sodium salt on the sorption capacities of the biocarbons was determined (BlueLine 25 pH electrode (SI Analytics, Weilheim, Germany)). Measurements were performed for 25 mg of the solid sample with 0.050 L of the MR solution of 40 mg/L concentration. The sorption studies of MR on the biocarbons obtained were also carried out at different temperatures. The biocarbon samples were prepared in the same way as for the study of the effect of pH on the adsorption capacities (25 mg of biocarbons and 40 mg/L of MR). We also studied the effect of the size of adsorbent portions used on their sorption capacities. Bottles were charged with different portions of the biocarbons studied, 15, 25 and 40 mg, and the biocarbons were flooded with 0.050 L of the dye solution (40 mg/L). Samples were shaken for 6 h on a shaker (Heidolph, Schwabach, Germany) at a rate of 200 rpm/min. After this time, spectrophotometric measurements were performed.

The kinetics of MR adsorption was analyzed using two Equations (2) and (3):

(2) $${\mathrm{logq}}_{e}={\mathrm{logK}}_{F}+\frac{1}{n}{\mathrm{logC}}_{e}$$

(3) $$\frac{t}{q_{t}}=\frac{1}{k_{2}q_{e}{}^{2}}+\frac{t}{q_{e}}$$

where: q_t_is the amount of MR adsorbed at time t [mg/g]; k_1_is the pseudo-first-order model rate constants of adsorption [1/min]; k_2_is the pseudo-second-order model rate constants of adsorption [g/mg × min].

The sorption studies of MR on the biocarbons obtained were also carried out at three different temperatures (25, 45, and 65 °C).

The experimental data were fitted to three models:Langmuir (4), Freundlich (5) and Temkin (6):

(4) $$q_{e}=\frac{q_{m}\times K_{L}\times C_{e}}{1+(K_{L}\times C_{e})}$$

(5) $$q_{e}=K_{F}\times C_{e}{}^{1/n_{F}}$$

(6) $$q_{e}={\mathrm{BlnA}}_{T}+{\mathrm{BlnC}}_{e}$$

where: q_m_ is the monolayer capacity [mg/g]; K_L_ is the Langmuir constant [L/mg]; K_F_ is the Freundlich constant [mg/g × (mg/L)1/n]; n_F_ is a constant that characterizes the surface heterogeneity; B is a constant equal to B = R_T_/B_T_, where B_T_ is the Temkin constant [J/mol], R is the universal gas constant [J/mol × K], T is the absolute temperature [K], and A_T_ is the Temkin isotherm equilibrium binding constant [L/mg].

## 3. Results

### 3.1. Characterization of Biocarbons

The main component of the samples obtained was elemental carbon (Table 1). The content of this heteroatom varied from 69.1 to 82.2 wt.%. An increase in the activation temperature by 100 °C brought about a decrease in the content of carbon and a significant increase in the content of oxygen in the structure of the obtained adsorbents. As to the contents of hydrogen and nitrogen, the increase in the activation temperature led to a slight reduction in their contents. The content of mineral matter in the obtained biocarbons varied from 7.8 to 11.2 wt.%. This is relatively low as, according to the literature data, the biocarbon adsorbents obtained by direct activation of plant materials usually contain large amounts of mineral matter admixtures [33,34].

Table 2 presents the textural parameters of the biocarbons obtained. It should be noted that the activation of fennel and caraway fruits with carbon(IV) oxide in a microwave oven does not lead to the effective development of the porous structure of the obtained biocarbons. As seen in Table 2, S_BET_ of the biocarbons depended on the starting material and activation temperature. The greatest specific surface area was determined for sample FA8 (371 m^2^/g), obtained as a result of the physical activation of fennel fruit at 800 °C. However, the values of specific surface areas of the adsorbents studied were similar and the differences in this parameter were small, not exceeding 100 m^2^/g.

**Figure 1:**
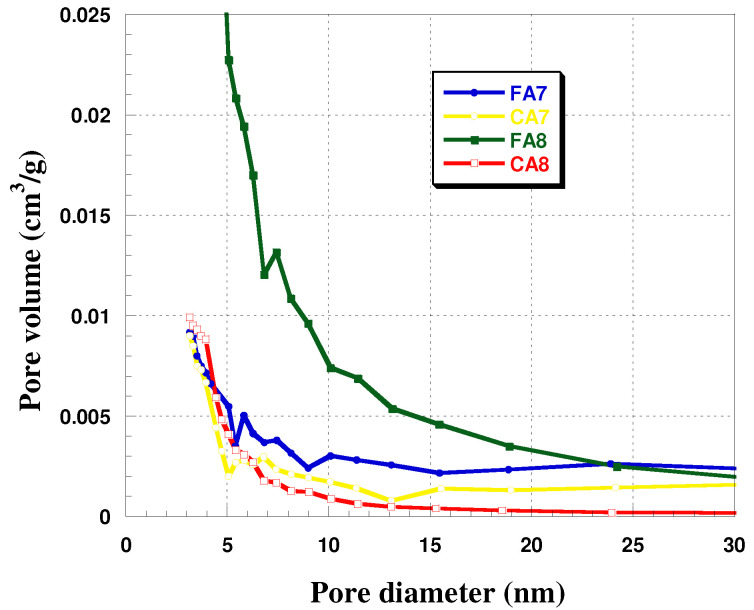
Pore size distribution of samples obtained.

**Figure 2:**
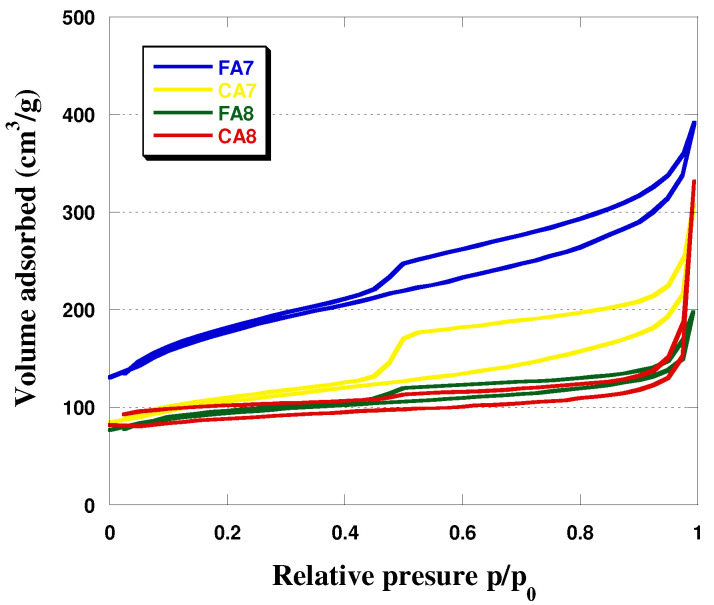
Low-temperature nitrogen adsorption/desorption isotherms of samples obtained.

Analysis of the effects of the type of starting material and activation temperature shows that with increasing temperature of activation, the specific surface area of the biocarbons increased. Moreover, greater S_BET_ was obtained for the biocarbons obtained from fennel fruit. The activation temperature was also observed to have impact on the mean pore size in the biocarbons studied (Figure 1, Table 2). For samples FA7 and CA7, the average pore sizes were 6.37 and 7.65 nm, respectively, while for samples FA8 and CA8 (activated at 800 °C), the analogous values were 4.19 nm and 3.68 nm. As for the contribution of micropores in the sample structures, it was the greatest—63%—for CA8. The character of the structure of the biocarbons studied was corroborated by isotherms, presented in Figure 2. For FA7 and CA7, a distinct hysteresis loop of H4 type was observed, whose presence indicates a greater contribution of mesopores in the porous structures of these biocarbons. The isotherms recorded for FA8 and CA8 also show hysteresis loops, but much smaller ones, which implies that their pores were a.

The iodine numbers determined for the obtained biocarbons are presented in Table 2. Adsorption of iodine water solution on carbon adsorbents permits characterization of the ability of the latter to remove inorganic pollutants of molecule diameters close to 1 nm [35]. The obtained biocarbons showed low sorption capacity towards iodine in water solution. It is a consequence of poorly developed S_BET_ and, more importantly, porous structure of the adsorbents based on fennel and caraway fruits, activated by carbon(IV) oxide. The most effective adsorbent proved to be biocarbon FA8, that adsorbed 545 mg of iodine. The iodine number of the studied samples was found to depend on temperature, irrespective of the type of precursor; the effectiveness of iodine adsorption of the obtained biocarbons increased with increasing temperature. For the samples obtained from fennel fruit, an increase of 201 mg was noted, while for those obtained from caraway, the increase was of 186 mg, upon increasing the activation temperature by 100 °C.

**Figure 3:**
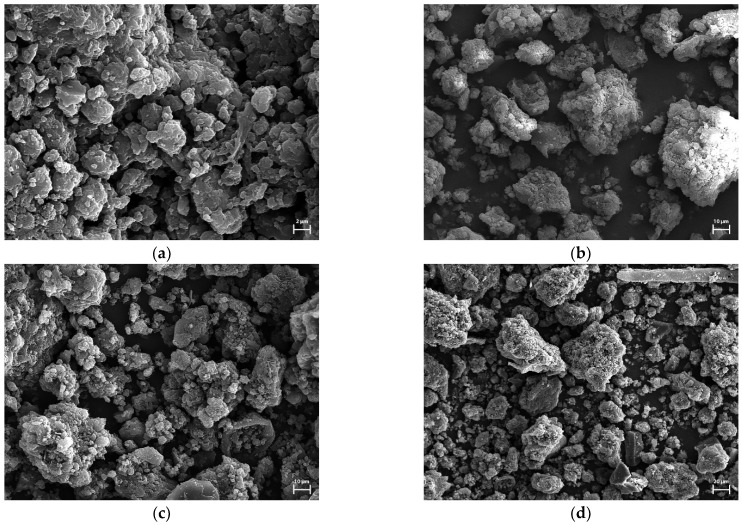
SEM micrographs of FA7 (**a**), CA7 (**b**), FA8 (**c**) and CA8 (**d**) prepared.

The SEM micrographs of the obtained biocarbons are presented in Figure 3. The brighter fragments observed on the biocarbons may be due to the presence of ash content.

On the basis of the results presented in Table 2 and those reported in [33,34,36], it can be concluded that direct activation with carbon(IV) oxide of the biomaterials studied does not lead to development S_BET_ of the obtained bioadsorbents; however, this does not mean that these materials have no potential for application in the removal of organic and inorganic pollutants from the liquid or gas phase. Kazmierczak et al. [33] have used bio-activated carbons prepared by direct activation of hay with the use of microwave radiation for adsorption of gas (NO_2_) and liquid pollutants (iodine, methylene blue, Congo red). The biocarbon adsorbents they obtained showed a specific surface area, not exceeding 300 m^2^/g. These authors compared their results with the literature data and indicated that low-quality hay can be used as starting material for obtaining samples, showing high sorption capacity towards gas and liquid pollutants. Nowickiet al. [36] have demonstrated that activated biocarbons obtained as a result of direct physical activation and chemical activation of post-fermentation residue, despite poorly developed surface area (21–877 m^2^/g), show a comparable or sometimes higher sorption capacity towards methylene blue and malachite green in water solutions than the commercially available Norit^®^ SX2 carbon.

Chemical character of the obtained biocarbons was determined by Boehm titration. Moreover, the acid-base properties of the samples were confirmed by measurement of pH of the water extracts of these adsorbents (Table 3).

The biocarbons obtained show similar acid-base properties; that is, their surfaces have distinctly basic character. The content of functional groups of basic character varied in the range 2.34–4.05 mmol/g, while that of the oxygen functional groups of acidic character varied from 0.05 to 0.25 mmol/g. Results of the Boehm titration for samples FA7, CA7, FA8 and CA8 were confirmed by the pH values of the samples of water extracts, that fell in the range 9.33–10.46. Higher pH values were obtained for the samples activated at 800 °C. Direct activation of plant origin precursors led to bioadsorbents of clearly basic surface character. Generation of basic groups is promoted by the presence of carbon(IV) oxide used as an activator and a high temperature of activation [31,34].

### 3.2. Adsorption Study

#### 3.2.1. Adsorption Equilibrium

**Figure 4:**
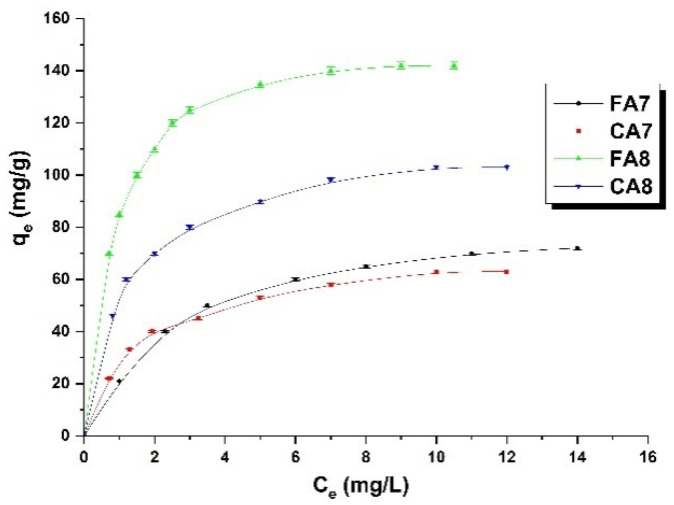
Isotherms of methyl red sodium salt adsorption on biocarbons (biocarbon mass: 25 mg, initial dye solution concentration: 10–80 mg/L, methyl red sodium salt solution volume: 50 mL, temperature: 23 ± 1 °C).

Figure 4 presents the relationship between the concentration of methyl red sodium salt in water solution and the sorption capacity of the prepared samples. As seen in this figure, with increasing concentration of the dye, the sorption capacity of the samples studied increases. This relation held for all adsorbents, which means that with increasing concentration of the dye, the effectiveness of the interactions/collisions between the adsorbate and adsorbent increases, leading to increased sorption capacity [15]. The sorption capacities of the samples studied were in the range of 63 to141 mg/g. The capacities of samples FA8 and CA8 were much greater than those of their analogues activated at 700 °C; for FA8 the increase was 70 mg, while for CA8 it was 42 mg. The greater sorption capacities of samples FA8 and CA8 were related to their slightly better-developed surface area.

#### 3.2.2. Adsorption Isotherms

**Figure 5:**
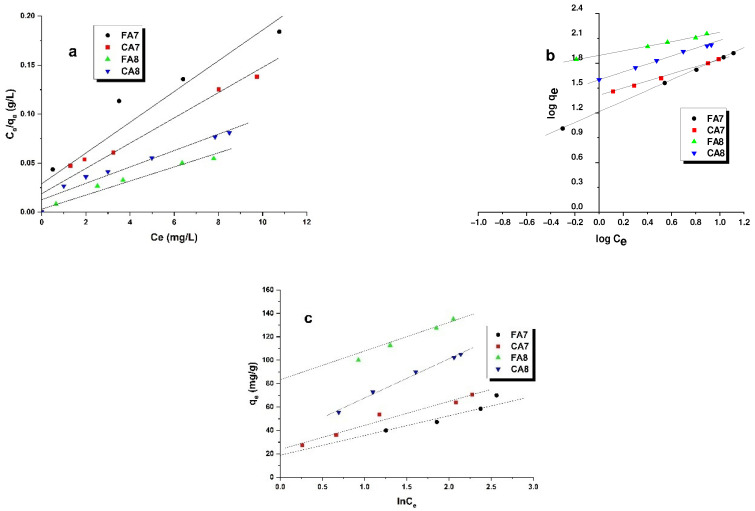
Linear fitting of methyl red sodium salt adsorption isotherms on biocarbon adsorbents to Langmuir (**a**), Freundlich (**b**) and Temkin (**c**) models.

The experimental data were fitted by Langmuir, Freundlich and Temkin models (Table 4, Figure 5). The best fit was obtained for the Freundlich model, which indicates that the interactions between methyl red sodium salt and the adsorbents obtained involve chemisorption. This model assumes multilayer adsorption, in contrast to monolayer formation as in the Langmuir model, made of layers of inhomogeneous heat distribution because of the heterogeneity of the active centers on the biocarbons obtained. The type of isotherm was inferred from the value of parameter n that, similarly to the K_F_ constant, depended on temperature [37]. The value of 1/n determined for the biocarbons obtained varied in the range 0.277–0.547, which indicates that the chemical bonds formed between the biocarbons and methyl red sodium salt are strong [38].

The biocarbons activated with carbon(IV) dioxide at 800 °C were more selective towards methyl red sodium salt than samples FA7 and CA7; for FA8 and CA8, the K_F_ values were 78.16 and 39.63 mg/g(L/mg)^1/n^, respectively. The reaction of the methyl red sodium salt from the water solution on the surfaces of the biocarbons obtained was inferred to have exothermal character, as the value of B for the Temkin model was higher than 0 [39]. The theoretically found maximum sorption capacity g_max_ was the closest to the experimental data obtained for the samples based on fennel fruit as a precursor.

#### 3.2.3. Adsorption Kinetics

**Figure 6:**
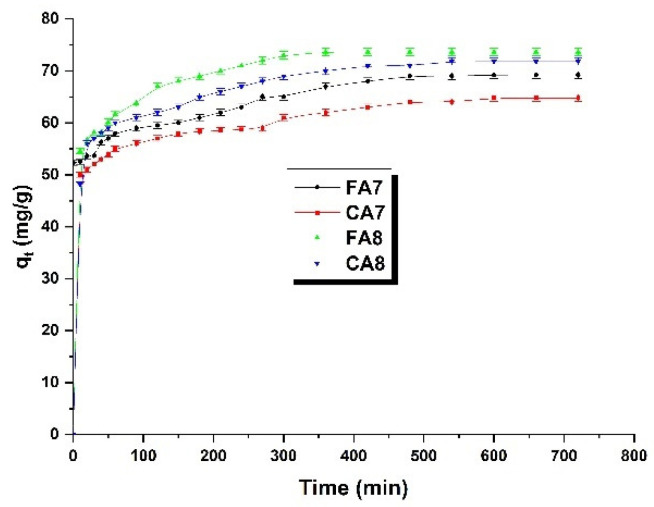
The effect of contact time of biocarbon with methyl red sodium salt (biocarbon mass: 25 mg, initial dye solution concentration: 40 mg/L, volume of methyl red sodium salt solution: 50 mL, temperature: 23 ± 1 °C).

The next objective of our study was to determine the effect of contact time of methyl red sodium salt with the biocarbons studied on the character of adsorption. The outcomes are presented in Figure 6, and the experimental data permitted determination of parameters of two kinetics models (Table 5).

Methyl red sodium salt adsorption from water solution was very fast at the beginning of the process. In the first phase of the process, the surfaces of all samples studied, FA7,CA7, FA8 and CA8, had a significant number of active centers easily accessible to the dye [40], which led to a rapid augment in sorption capacity. As the process time increased, the active centers on the sample surface were occupied and a state of adsorption equilibrium is reached. As seen in the shape of the isotherms, the adsorption equilibrium was achieved for all samples in about 500 min. After this time, no further increase in sorption capacities was observed. The time needed to reach the adsorption equilibrium by the samples studied was close to 8 h, which is rather long taking into account the results presented in [15,41].

**Figure 7:**
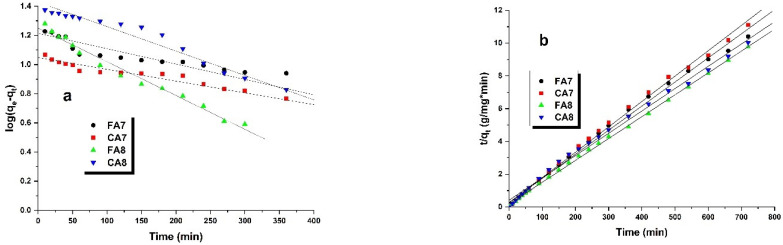
Pseudo-first-order (**a**) and pseudo-second-order (**b**) kinetic plots for adsorption of methyl red sodium salt onto biocarbons.

The fit of the data obtained in this study to the pseudo-first-order and pseudo-second-order kinetic models provides information on the quantitative character and mechanism of adsorption [42]. Analysis of Table 5 and Figure 7 indicates that the kinetics of adsorption of methyl red sodium salt from aqueous solution on biocarbon surface samples studied is better described by the pseudo-second-order-model, as proven by higher values of the correlation coefficient R^2^. Moreover, the value of q_e(cal)_ for this model is close to that of q_t(exp)_ obtained for biocarbon samples studied. Thus, it can be inferred that the adsorption of the dye on the test samples is controlled by a chemical process [40].

#### 3.2.4. The Effect of pH

**Figure 8:**
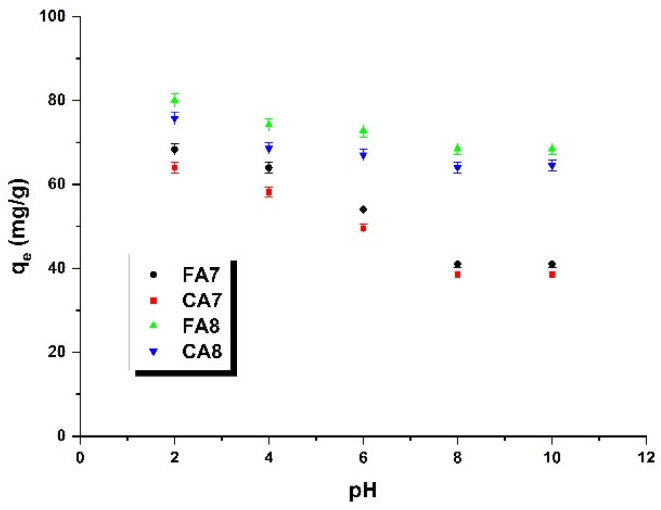
The pH influence on the adsorption of methyl red sodium salt (biocarbon mass: 25 mg, initial dye solution concentration: 40 mg/L, methyl red sodium salt solution volume: 50 mL, temperature: 23 ± 1 °C).

Figure 8 illustrates the impact of the pH of the dye solution (a) and adsorption temperature (b) on the efficiency of removal of methyl red sodium salt from the aqueous solution by the biocarbons obtained from fennel and caraway fruits as precursors, activated by carbon(IV) oxide. The effect of the pH of the dye solution was analyzed in the pH range of 2 to 12. According to the data presented in Figure 8, as the sorption capacity of all samples decreased, the pH of the dye solution increased, so the supreme sorption capacities were noted at a pH of 2. Then, the sorption capacities gradually decreased with pH increasing up to a pH of 8. At a pH of 10, no changes were noted. For samples FA7 and CA7, the effectiveness of the dye adsorption decreased much faster than for FA8 and CA8. The gradual decrease in sorption capacity with increasing pH of the methyl red sodium salt solution may be a consequence of the electrostatic interaction occurring between the anion dye and the adsorbent surface, as the acidic groups present in the biocarbon samples may interact with the dye molecules [43].

#### 3.2.5. Thermodynamic Study

**Figure 9:**
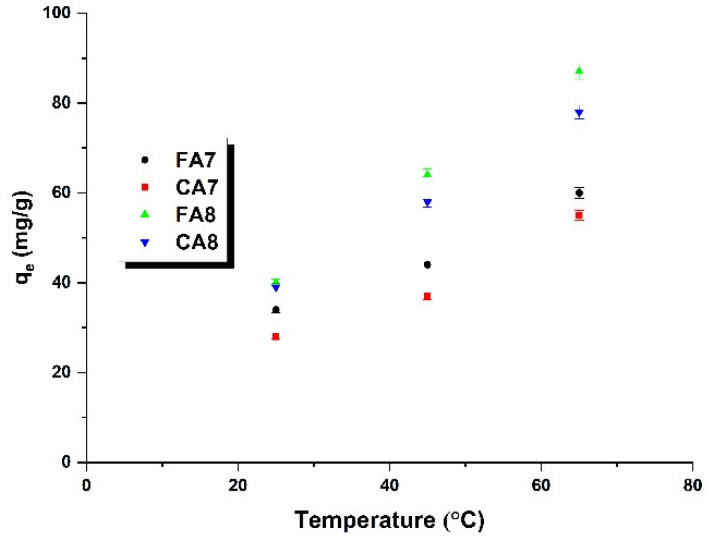
The temperature influence on the adsorption of methyl red sodium salt (biocarbon mass: 25 mg, concentration of initial dye solution: 40 mg/L, methyl red sodium salt solution volume: 50 mL).

The effect of adsorption temperature on the sorption capacities of the biocarbon adsorbents was tested in the range of 25 to 65 °C, and the results are presented in Figure 9 and Table 6. As displayed in Figure 9, the greatest sorption capacities were measured for the process taking place at 65 °C. Moreover, as the adsorption temperature increased, the sorption capacities of all samples increased, which suggests that the process of adsorption has an endothermic character [44]. Analysis of the data presented in Figure 9 indicates that temperature of adsorption has no significant effect on the dye removal efficiency. For sample FA7, the increase in sorption capacity reaches about 25 mg, while for the other bioadsorbents the capacity was about 40 mg.

Knowledge of the effect of temperature on dye removal efficiency permits determination of the following three thermodynamical parameters [36]: Gibbs free energy (ΔG), enthalpy (ΔH), and entropy (ΔS, Table 6), from the equations given below (7) and (8):

(7) $$\log(\frac{q_{e}}{C_{e}})=\frac{\Delta S}{2.303R}-\frac{\Delta H}{2.303\mathrm{RT}}$$

(8) ΔG=ΔH−TΔS

where: R = gas constant–8.314 [J/K×mol]; T = temperature [K]. The values of ΔH and ΔS can be obtained from the slope and intercept of (log(q_e_-C_e_) vs. 1/T).log(q_e_/C_e_) vs. 1/T

As the Gibbs free energy values vary in the range of−20 to 0 kJ/mol, the adsorption process has a physical character [45]. Moreover, with the increasing temperature of the process, the values of ΔG become more negative, which indicates an increase in the spontaneity of the process. Positive values of ΔH confirm that the process of adsorption of methyl red sodium salt from aqueous solution on the tested biocarbons was endothermic and requires energy expenditure [46].

#### 3.2.6. Effect of Adsorbent Amounts

**Figure 10:**
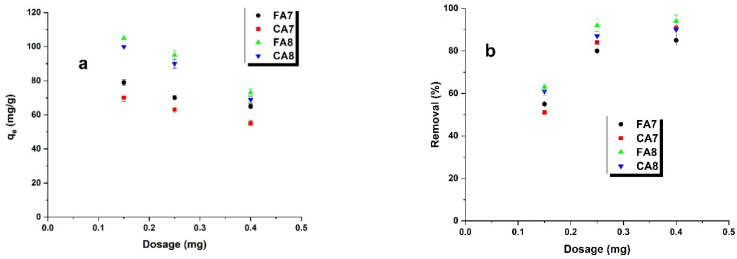
Effect of dosage (**a**) and adsorption efficiency (**b**) on the adsorption of methyl red sodium salt on biocarbons.

Figure 10a presents the effects of the amounts of adsorbents (or portion size of the adsorbent) on their sorption capacities towards methyl red sodium salt in water solutions. The adsorbent doses varied from 0.3 to 0.8 g/L. Analysis of the results shows that the use of an increasing amount of adsorbents (or an increasing size of the adsorbent portions) leads to decreasing sorption capacities. This is a consequence of a decreased amount of the dye per unit mass of the adsorber, which leads to a diminished coefficient describing the use of active sites. The effectiveness of the dye removal from its water solution (Figure 10b) increased with increasing size of the adsorbent portions, so with increasing surface area and number of active sites [47]. For the adsorbent portions of 0.025 and 0.040 g, no significant changes were noted.

#### 3.2.7. Desorption

**Figure 11:**
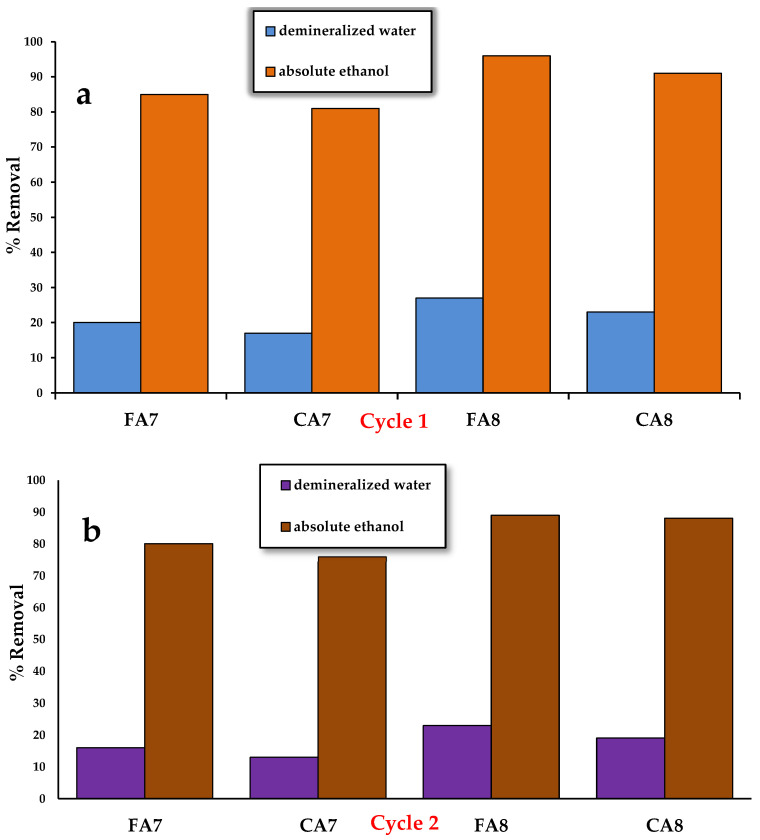
Effect of different eluents on the desorption of methyl red sodium salt, cycle 1 (**a**) and cycle 2 (**b**).

The possibility of the reuse of biocarbon in adsorption is of great importance [37]; therefore, at the next stage of the study, desorption of methyl red sodium salt from the biocarbon’s surface was performed. Portions of 0.050 g of each adsorbent were flooded with 50 mL of a dye solution with a concentration of 40 mg/L. The samples were shaken for 12 h on a shaker (Heidolph, Schwabach, Germany) at a rate of 200 rpm/min. After drying, the biocarbons were flooded with 10 mL of two eluents: demineralized water and absolute ethanol. The methyl red sodium salt was washed out for 6 h and the amount of the desorbed dye was determined by the spectrophotometric method at 443 nm. The changes in the sorption capacity of the biocarbons after one (Figure 11a) and two (Figure 11b) cycles of regeneration showed that the desorption was more effective when using absolute ethanol.

#### 3.2.8. Adsorption Capacities of Selected Materials

Methyl red belongs to the group of mono-azo anionic dyes and is widely applied in the textile industry [48]. It has mutagenic effects on living organisms in aerobic conditions [49]; therefore, much attention is being paid to finding effective methods of removing it from aqueous solutions before releasing into environment. Table 7 presents the sorption capacities of adsorbents used for the removal of this dye and the main conclusions from our studies.

Comparing the sorption capacity obtained for the biocarbon obtained from fennel, sample FA8, and the other results listed in Table 7, it is apparent that the former is much greater than the sorption capacities of the bark of the *D. viscosa* tree [43], banana pseudostem fibers [50], and activated carbon obtained from *Annona squamosa* seeds [15]. The adsorbents received from the above-mentioned bark and banana fibers have not been subjected to any thermochemical treatment, which is undoubtedly to their advantage, but nevertheless, their sorption capacities are lower than that of FA8. Moreover, the cost of production of biocarbon from fennel used as a precursor was minimized by the application of direct physical activation with CO_2_ and microwave heating. The cost of this process is certainly lower than that of the synthesis of oxidized multiwalled carbon nanotubes [51]. The sorption capacity of the activated carbon prepared from Annona squamosa seeds, of 27.68, was also much lower than that of FA8 [16]. The former material was also prepared with no thermochemical treatment; *Annona squamosa* seeds were flooded with concentrated H_2_SO_4_ for 12 h, then washed with distilled water and flooded with 2% sodium bicarbonate to remove the excess acid. Adsorption of methyl red on the activated carbon, prepared from residues (*Annona squamosa seeds)*, was described by the pseudo-second-order model, similarly to that on FA8, but an adsorption monolayer was formed on the surface of the adsorbent.

**Figure 12:**
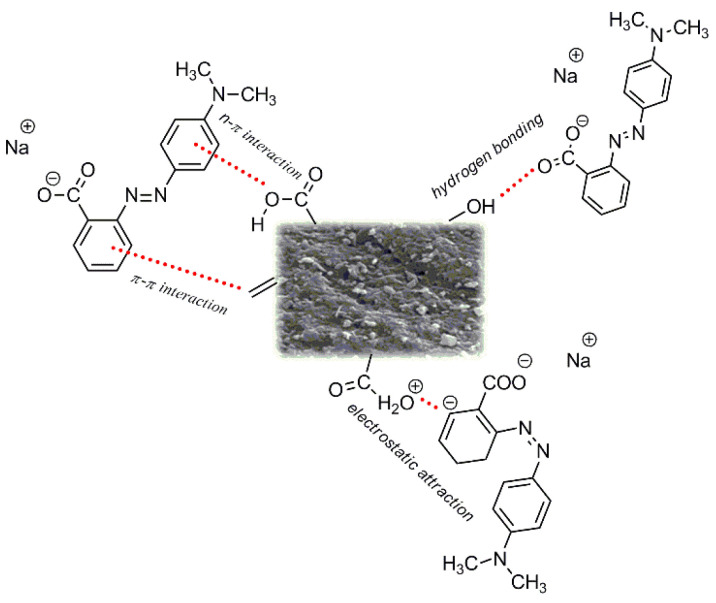
The adsorption mechanism of methyl red sodium salt by biocarbons obtained.

Much greater effectiveness in removal of methyl red was seen with the activated carbon obtained from custard apple (*Annona squamosa*) fruit shell [35]; its sorption capacity was over 100 mg/g greater than the capacity of biocarbon FA8. The adsorbent based on *Annona squamosa* fruit shell [35] was obtained by chemical activation using phosphoric acid at a weight ratio of the precursor to activator 1:1.5. After activation, the adsorbent was washed with a 1% solution of sodium bicarbonate and doubly distilled hot water, which increased the production cost of these bioadsorbents. The carbon sample obtained from the *Annona squamosa* fruit shell showed the specific surface area of 1064.95 m^2^/g, which is much greater than that of sample FA8’s 371 m^2^/g. Moreover, the much better developed porous structure of the carbon adsorbent obtained by chemical activation also contributed to its high sorption capacity. It may be expected that the increase in the activation temperature and/or time of activation for the biocarbon based on fennel fruit would result in obtaining samples of greater specific surface area and, thus, higher sorption capacity. That is why in subsequent studies attention will be paid to the proper choice of these parameters in the process of biocarbon synthesis. Figure 12 presents the possible interactions that may take place between the methyl red molecules and the surface of biocarbons. The sorption capacities obtained in this study may be related to electrostatic interactions and the formation of hydrogen bonds. The mechanism of adsorption of methyl redsodium salt may also involve the π-π and n-π interactions. It should be highlighted that the adsorption of methyl red sodium salt may be affected not only by the structure of the dye, but also the by the properties of the surface of carbon adsorbents.

## 4. Conclusions

This paper presents the results of the study on bioadsorbents obtained from fennel and caraway fruits by activation with carbon(IV) oxide, using microwave radiation as a source of heat. The obtained biocarbons were found to show poorly developed porous structure and a surface of definitely basiccharacter. The specific surface area and chemical character of the samples were significantly dependent on the type of precursor and the temperature of activation. Moreover, an increase in the temperature of activation led to increase in the contents of carbon, oxygen and ash in the structure of the biocarbons obtained. The process of adsorption on the biocarbons studied was proven to be best described by the Freundlich model, which means that the adsorbed substance makes multilayers on the adsorbent surface The kinetics of the process was best described by the pseudo-second-order equation. Thus, it can be concluded that the adsorption of the methyl red sodium salt on the biocarbons studied is controlled by a chemical process. In order to reach the state of adsorption equilibrium, the process of dyeing should be performed for 8 h. Moreover, low pH of the dye solvent had positive impact on the sorption capacities of the biocarbon adsorbents studied. With increasing temperature, the sorption capacities of the biocarbon adsorbents towards methyl red sodium salt increased. According to the thermodynamical parameters of the process, the process has an endothermic and spontaneous character. Results of the desorption study of methyl red sodium salt from the surface of the biocarbons studied has confirmed that the latter can be successfully reused.

## Figures and Tables

**Table 1:** Elemental composition of the adsorbents obtained [wt.%].

Sample	Ash	C	H	N	O *
FA7	9.0	73.0	1.9	0.9	24.2
CA7	7.8	82.2	1.2	0.8	15.8
FA8	11.2	69.1	1.8	0.8	28.3
CA8	8.5	72.0	1.1	0.8	26.1

*—By difference; method error ≤0.3%.

**Table 2:** Textural parameters and iodine number of the samples obtained.

Biocarbons	Surface Area ^1^ (m^2^/g)	Total Pore Volume (cm^3^/g)	Micropore Area (m^2^/g)	Average Pore Diameter (nm)	Iodine Number (mg/g)
**FA7**	295	0.20	282	6.37	345
**CA7**	233	0.51	222	7.65	303
**FA8**	371	0.61	360	4.19	545
**CA8**	330	0.48	223	3.68	489

^1^ Error range between 2–5%.

**Table 3:** Acid-base properties of the biocarbons obtained.

Biocarbons	Acidic Oxygen Functional Groups (mmol/g)	Basic Oxygen Functional Groups (mmol/g)	pH
**FA7**	0.25 ± 0.01	2.67 ± 0.02	9.56 ± 0.01
**CA7**	0.19 ± 0.01	2.34 ± 0.02	9.33 ± 0.01
**FA8**	0.05 ± 0.01	4.05 ± 0.03	10.46 ± 0.01
**CA8**	0.09 ± 0.01	3.99 ± 0.03	10.00 ± 0.01

**Table 4:** The parameters calculated from Langmuir, Freundlich and Temkin models.

Sample	Langmuir			Freundlich			Temkin		
	q_m_ (mg/g)	K_L_ (L/mg)	R^2^	K_F_ (mg/g(L/mg)^1/n^)	1/n	R^2^	A_T_ (L/mg)	B	R^2^
**FA7**	71.43	0.008	0.900	16.48	0.547	0.996	3.07	16.81	0.918
**CA7**	76.92	0.013	0.949	25.89	0.430	0.981	3.18	20.52	0.968
**FA8**	142.86	0.025	0.976	78.16	0.277	0.993	4.53	39.15	0.972
**CA8**	125.00	0.007	0.945	39.63	0.477	0.995	3.12	32.26	0.979

**Table 5:** Kinetic models parameters.

Sample	q_t(exp)_ (mg/g)	Pseudo-First-Order-Model			Pseudo-Second-Order-Model		
		q_e(cal)_ (mg/g)	k_1_(1/min)	R^2^	q_e(cal)_ (mg/g)	k_2_ (g mg^−1^×min^−1^)	R^2^
**FA7**	69.20	16.37	0.0023	0.916	71.42	0.0006	0.994
**CA7**	64.77	11.17	0.0002	0.963	66.67	0.0008	0.996
**FA8**	73.65	17.83	0.0046	0.980	76.92	0.0009	0.999
**CA8**	71.81	26.79	0.0023	0.976	74.63	0.0004	0.994

**Table 6:** Thermodynamic parameters.

Sample	Temperature (°C)	Gibbs Free Energy (kJ × mol^−1^)	Enthalpy (kJ × mol^−1^)	Entropy (J × mol^−1^ × K^−1^)
**FA7**	25	−2.19	16.85	63.68
	45	−3.32		
	65	−4.77		
**CA7**	25	−1.90	17.35	65.05
	45	−2.98		
	65	−4.52		
**FA8**	25	−4.32	23.34	92.87
	45	−6.26		
	65	−8.03		
**CA8**	25	−5.09	25.18	100.77
	45	−7.07		
	65	−9.16		

**Table 7:** Comparison of adsorption capacity with various reported adsorbents for methyl red removal.

Adsorbent	Adsorption Capacity (mg/g)	Isotherm	Kinetics	Thermodynamics	References
Activated carbon from custard apple (*Annona squamosa*) fruit shell	243.5	Langmuir	pseudo-first-order	spontaneous, endothermic	[15]
Bark of the *D. viscosa tree*	36.64	Freundlich	-	-	[43]
Banana pseudostem fibers	88.50		pseudo-second-order	-	[50]
Activated carbon prepared from *Annona squamosa* seeds	27.68	Langmuir	pseudo-second-order	-	[16]
Oxidized multiwalled carbon nanotubes	108.69	Langmuir	pseudo-first-order	spontaneous, endothermic	[51]
FA8	141	Freundlich	pseudo-first-order	spontaneous, endothermic	This study

## Data Availability

Data is contained within the article.
